# Saliva as a non-invasive specimen for COPD assessment

**DOI:** 10.1186/s12931-022-01935-9

**Published:** 2022-01-29

**Authors:** Sara Melo-Dias, Carla Valente, Lília Andrade, Alda Marques, Ana Sousa

**Affiliations:** 1 Department of Medical Sciences, University of Aveiro, Aveiro, Portugal; 2 Lab3R-Respiratory Research and Rehabilitation Laboratory, School of Health Sciences (ESSUA), University of Aveiro, Aveiro, Portugal; 3 Institute of Biomedicine (iBiMED), University of Aveiro, 3810-193 Aveiro, Portugal; 4 Department of Pulmonology, Hospital Center of Baixo Vouga, Aveiro, Portugal

**Keywords:** Microbiota, Biomarker, COPD, Salivary bacteria, Respiratory diseases, Microbiome

## Abstract

**Background:**

People with COPD have been reported to bear a distinct airway microbiota from healthy individuals based on bronchoalveolar lavage (BAL) and sputum samples. Unfortunately, the collection of these samples involves relatively invasive procedures and is resource-demanding, limiting its regular use. Non-invasive samples from the upper airways could constitute an interesting alternative, but its relationship with COPD is still underexplored. We examined the merits of saliva to identify the typical profile of COPD oral bacteria and test its association with the disease.

**Methods:**

Outpatients with COPD and age-sex matched healthy controls were recruited and characterised based on clinical parameters and 16S rRNA profiling of oral bacteria. A clustering analysis based on patients’ oral bacteria beta-diversity and logistic regressions were performed to evaluate the association between oral bacteria composition and COPD.

**Results:**

128 individuals participated (70 patients and 58 controls). Differential abundance analyses showed differences in patients comparable to the ones previously observed in samples from the lower respiratory tract, *i.e*., an increase in Proteobacteria (particularly *Haemophilus*) and loss of microbiota diversity. An unsupervised clustering analysis separated patients in two groups based on microbiota composition differing significantly in the frequency of patients hospitalized due to severe acute exacerbation of COPD (AECOPD) and in the frequency of GOLD D patients. Furthermore, a low frequency of *Prevotella* was associated with a significantly higher risk of recent severe AECOPD and of being GOLD D.

**Conclusion:**

Salivary bacteria showed an association with COPD, particularly with severe exacerbations, supporting the use of this non-invasive specimen for future studies of heterogeneous respiratory diseases like COPD.

**Supplementary Information:**

The online version contains supplementary material available at 10.1186/s12931-022-01935-9.

## Background

The respiratory physiology of patients with chronic obstructive pulmonary disease (COPD) hamper mucociliary clearance in the airways which leads to an exceptional opportunity for bacterial proliferation [1] and results in the establishment of a resident community [2].

In accordance, patients with COPD have been reported to bear a distinct airway microbiota from healthy individuals based on bronchoalveolar lavage (BAL) and sputum specimens [3], though a “typical” COPD profile is difficult to assign since it continuously modifies with disease progression [4]. Nevertheless, some consensus exists regarding (i) a positive correlation between disease severity and microbiota composition, e.g., more severe patients are enriched in Proteobacteria (particularly *Haemophilus*) [5–8] and (ii) a negative correlation between disease severity and microbiota diversity [4, 5, 9].

However, evidence for clinical implications of these changes in COPD is still lacking, need short and long-term validation but is fundamental as these might be a promising biomarker of the disease.

Unfortunately, induced sputum or BAL collection are relatively invasive and resource-demanding procedures to be routinely performed (e.g. weekly), requiring trained health-care professionals and specialized equipment. Bronchoscopy in patients with COPD carries a significantly higher risk of complications such as pneumonia, respiratory failure and desaturation compared with those with normal lung function [10].Induced sputum collection, although semi-invasive, generally safe and well tolerated, may lead, especially in more debilitated patients, to some discomfort in sample collection [11, 12].

An interesting alternative would be the use of non-invasive specimens from upper airways, e.g. saliva, since the microbiota of upper and lower airways is highly correlated, and shows topological continuity, implying oral bacteria as the major colonizers of the lower airways, through microaspiration [13–15]. Consequently, both niches present several overlapping bacterial genera, e.g. *Prevotella*, *Veillonella* and *Streptococcus*, yet the microbiota from lower airways is less diverse and numerous [13–17]. Saliva’s collection is also friendly enough to be performed frequently (e.g., weekly) even in more debilitated patients.

Here, we have explored, for the first time, the merits of saliva, to identify the typical profile oral bacteria in stable COPD and to test its association with the disease. We have started by describing differences between groups of healthy and diseased individuals. Next, we queried the association between oral bacteria and COPD, by performing an unsupervised clustering analysis that allowed the stratification of people with COPD according to oral bacteria composition.

## Methods

A cross-sectional study was conducted. Ethical approvals were obtained from Administração Regional de Saúde Centro (64/2016) and from Centro Hospitalar do Baixo Vouga (08-03-17). Written informed consent was obtained from all participants. All steps of data collection, processing and analysis were summarized in the Additional  file 2.

### Subjects and sample collection

Participants with COPD and healthy (controls) were identified by physicians at primary health care centres, hospitals, or senior universities. Patients were eligible if (i) diagnosed with COPD according to the Global Initiative for Chronic Obstructive Lung Disease (GOLD) criteria [18], (ii) presented a stable state, with no acute exacerbations in the month prior to enrolment and (iii) were able to give informed consent. Exclusion criteria were (i) presence of severe cardiac, musculoskeletal, or neuromuscular diseases, (ii) cognitive impairment or (iii) active neoplasia or immune diseases. Healthy-individuals were age- and sex-matched to patients with COPD and had similar inclusion and exclusion criteria except for the absence of any respiratory disease. Sociodemographic, anthropometric and clinical data and saliva samples (passive drool) were collected with a structured protocol adapted from the team published work [19]. See Additional file 2 for further details upon data collection. GOLD grades were defined according to FEV1 percentage predicted for each individual. GOLD groups were defined combining the number of exacerbations and hospital admissions of each patient in the year before enrolment with their CAT scores.

### DNA extraction

DNA extraction from saliva samples was performed with QIAamp DNA Mini Kit (Qiagen, Hilden, Germany), following the manufacturer’s instructions with slight modifications. DNA quality and quantity was assessed in Denovix DS-11 spectrophotometer. See Additional file 2 for further details.

### 16S rRNA gene amplification and sequencing

V4 hypervariable region of 16S rRNA gene (F515/R806 primer pair) amplification and sequencing was carried out at the Gene Expression Unit from Instituto Gulbenkian de Ciência, according to the implemented protocol, using Illumina Miseq. See  Additional file 2 for further details.

### Oral bacteria and statistical analyses

#### Sample characterisation

Descriptive statistics was used to characterize the sample: comparisons between people with COPD and Healthy controls were conducted with unpaired t-test with Welch’s correction, Mann–Whitney U-test and Chi-square test (statistical analyses conducted in GraphPad Prism 8 [20] and *R* software v3.6.0 [21]). See Additional file 2 for further details.

#### Analysis of illumina paired-end reads

QIIME2 2020.8 [22, 23] was used to perform oral bacteria analyses. Quality control procedures were performed via q-score base filtering, chimera removing and 16S-denoising with Deblur [24]. Potential bacterial contaminants were identified with *DECONTAM* package [25, 26] of *R* [21] with prevalence method and excluded from subsequent analyses. Taxonomy assignment of amplicon sequence variants (ASVs) was performed with q2-feature-classifier plugin [27, 28], through classify-sklearn method with pre-trained Naïve Bayes classifier against 99% identity eHOMD_v15.1 reference database[29]. All subsequent analyses, except the differential abundance, were performed with data upon ASVs. Differential abundance analyses were done with data upon OTUs at taxonomic level 6 (genus).

#### Diversity analyses

Alpha-diversity metrics and Beta-diversity metrics were estimated using q2-diversity plugin [30] as implemented in QIIME2 [22, 23]. Spatial dissimilarities between bacterial communities of different groups were assessed with Principal Coordinate Analysis (PCoA) and/or biplots on Weighted Unifrac distance matrix. Mann–Whitney U-test and Kruskal–Wallis with Dunn’s correction were employed to compare alpha-diversity among groups (statistical analyses were performed in *GraphPad Prism 8* [20] and *R stats* package [31] of *R* [21]). Additionally, the effect of disease state (COPD vs healthy), and cluster segregation (cluster 1 vs cluster 2) on alpha diversity indexes was adjusted for pack-years using a Linear Regression Model (*R stats* package [31] of *R* [21]). Permutational multivariate analysis of variance (PERMANOVA) [32, 33] adjusted for pack-years (PY) (*vegan* package [34] of *R* [21]) was used to quantify the beta-diversity differences in oral bacteria composition of groups.

#### Differential abundance analysis of OTUs

Analysis of composition of microbiomes (ANCOM) [35, 36] and Linear discriminant effect size (LefSe) analysis [37, 38] were performed to identify differentially abundant operational taxonomic units (OTUs) between groups of samples and/or clusters. These analyses were conducted with the feature table collapsed at genus taxonomic level (L6). LEfSe was performed in the online version [38] with a linear discriminant analysis (LDA) score of 3 for significance. ANCOM was performed in R with ANCOM 2.0 script[36] with taxa-wise multiple correction and a W cut-off of significance of 0.7. See Additional file 2 for further details.

#### Clustering analysis

A hierarchical clustering analysis [39] of the oral bacteria (neighbour-joining) based on Weighted Unifrac distance was performed as implemented in QIIME2 [22, 23] under a rarefaction of 4000 sequences per sample and 5000 iterations. See Additional file 2 for further details. Mann–Whitney U-test and Chi-square test (*R stats* package [31] of *R* [21]), were used to describe differences in clinical features among different clusters.

#### Binary logistic regression models and ROC analyses

Binary logistic regression models (glm [40] (link = logit) function of *R stats* package [31] *R* software [21], adjusted for PY, were performed to further explore the relation between the most relevant ASVs/OTUs and clinical features in the context of the clustering analysis. Details upon models’ quality assessment were further described in supplementary file. Receiver operating characteristic curves (ROC) and respective discriminatory thresholds were estimated to assess the discriminatory ability of each model (*pROC* package [41] from *R* software [21]). Finally, the respective area under the curve (AUC) was also calculated for each model. See Additional file 2 for detailed description of the analyses performed.

## Results

### Cohort characterisation

Seventy people with COPD (60 male, 68 ± 9y, BMI 25.5 ± 3.5, FEV_1_pp 48 ± 16, GOLD A-12, B-32, C-5, D-21) and fifty-eight sex and age matched healthy individuals (42 male, 67 ± 8y, BMI 27.6 ± 3.8, FEV_1_pp 103 ± 17) were included in this study. Detailed characteristics of participants are available in Table 1 and Additional file 1: Table S1.

### Oral bacteria composition and diversity are different between people with COPD and healthy controls

Principal coordinate analysis of pairwise distances (Weighted Unifrac) between healthy and people with COPD showed significant differences in oral bacteria composition between groups (PERMANOVA adjusted for PY, *p* = 0.034) and captured 65% of total diversity (top three principal coordinates).

**Fig. 1:**
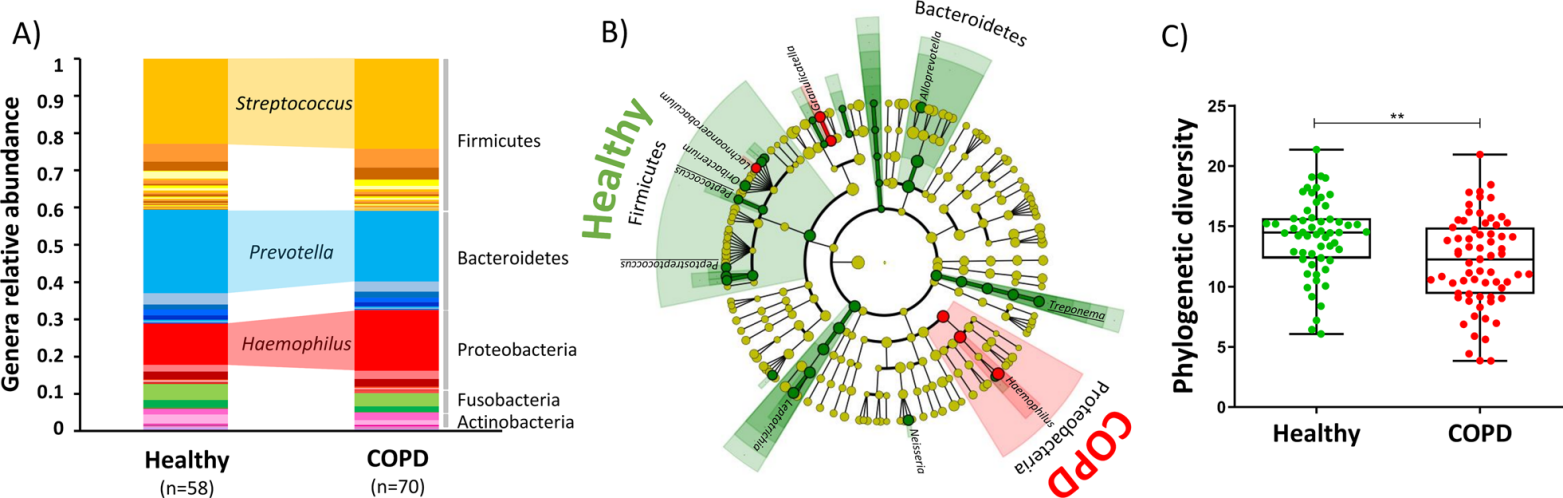
Salivary microbiota composition and diversity is different between people with COPD and healthy controls. **A** Mean frequency of phyla and genera of bacteria present in people with COPD and healthy controls. **B** Cladogram summarizing differentially abundant genera between people with COPD and healthy controls, assessed by LEfSe and ANCOM. Differential genera between groups identified only by LEfSe at a significance cut-off of 3 are represented in black, differential genera pointed by ANCOM at 0.7 significance cut-off are represented in underlined red **C** Alpha diversity, estimated with Faith’s phylogenetic diversity index, is lower in people with COPD than in healthy controls (Mann–Whitney U-test, U = 1275, p = 0.0013). *p < 0.05, **p < 0.01, ***p < 0.001, ****p < 0.0001

Oral bacteria of healthy individuals was composed of two major phyla, Firmicutes (40.6%) and Bacteroidetes (30.4%) (Fig. 1a). These were followed by Proteobacteria (16.3%), Fusobacteria (6.7%), Actinobacteria (2.5%) and six low abundant phyla (< 3.5%). In terms of genera, *Streptococcus* (23%), *Prevotella* (24%) and *Haemophilus* (11%) were the most abundant. People with COPD showed a similar oral bacteria composition to healthy individuals, however differences in the relative frequencies of Bacteroidetes (26.5%) and Proteobacteria (22.3%) were observed as well as in genera *Prevotella* (18%) and *Haemophilus* (15%).

Differential abundant bacterial groups between people with COPD and healthy individuals were inferred with LEfSe and ANCOM. Both methods showed that healthy individuals were enriched in *Treponema* (Spirochaetes), *Peptococcus* (Firmicutes) and *Peptostreptococcus* (Firmicutes), whereas according to LEfSe, patients were enriched in genera from Proteobacteria and Firmicutes. Specifically, people with severe airflow obstruction showed an enrichment in *Haemophilus,* while those with moderate airflow obstruction were enriched in *Granullicatella* and *Lachnoanaerobaculum* (see Additional file 2: Fig. S1 for the complete list of genera that differ between the groups).

Oral bacteria of people with COPD was significantly less diverse (Phylogenetic diversity—Alpha diversity, i.e., within individual diversity) than that of healthy individuals (Fig. 1c, Mann–Whitney U test, U = 1275, *p* = 0.0013). Similar differences were observed after adjusting for PY (ANOVA, F-value = 10.89, p = 0.002).

### Oral bacteria composition and diversity are poorly associated with clinical features

We next explored the relationship between oral bacteria and patients’ clinical features. Specifically, we queried whether different levels of airflow obstruction (GOLD grades) and severity of previous exacerbations and symptoms (GOLD groups) were associated with significant differences in oral bacteria diversity and composition.

Considering airflow obstruction, moderate patients (GOLD 1 & 2) showed a significantly distinct oral bacteria composition when compared with severe patients (GOLD 3 & 4) (PERMANOVA adjusted for PY, *p* = 0.002) but no significant differences were observed in alpha-diversity (Mann–Whitney U-test, U = 435, *p* = 0.12).

PCoA analysis separated A + B from C + D groups based on the severity of previous exacerbations but not A + C from B + D groups based on the severity of symptoms (PERMANOVA adjusted for PY (A + B vs C + D), *p* = 0.03, PERMANOVA adjusted for PY (A + C vs B + D), *p* = 0.06). Alpha-diversity was not significantly different among different levels of severity of previous exacerbations or symptoms (Mann–Whitney U test (A + B vs C + D), U = 420, *p* = 0.21; Mann–Whitney U test (A + C vs B + D), U = 392, *p* = 0.64).

No significant associations were found between alpha-diversity and pack-years, hospital admissions, long-term oxygen therapy, treatment with inhaled corticosteroids and SpO_2_ in people with COPD.

### Oral bacteria are associated with disease severity in people with COPD

**Fig. 2:**
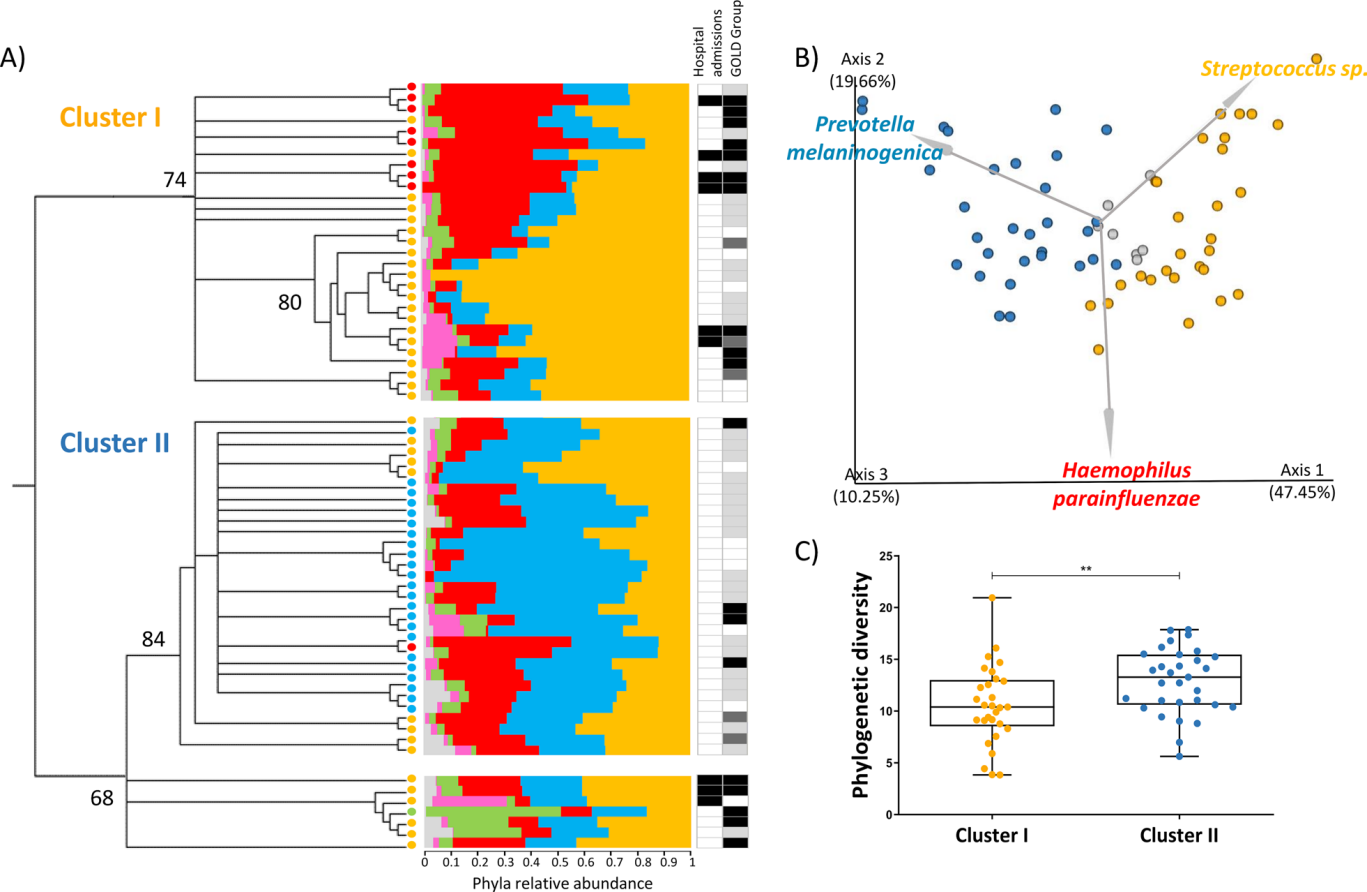
Unsupervised clustering analysis of the microbiota of people with COPD. **A** Dendrogram representing Neighbour joining clustering of Weighted Unifrac (samples rarefied by 4000 sequences, with 5000 iteractions). Numbers close to the internal nodes represent bootstrap support. Two major clusters containing 90% of people with COPD emerged: Cluster I and Cluster II. The bar chart represents microbiota composition of each patient at phylum level (Orange—Firmicutes; Blue—Bacteroidetes; Red—Proteobacteria; Green—Fusobacteria; Pink—Actinobacteria). Orange, blue, red and green circles represent the dominant phylum of each sample. The heatmap shows patient status according to “hospital admissions” and “Gold group”. Shading from white to black is proportional to severity level, white less severe and black most severe. **B** PCoA analysis using Emperor of Weighted UniFrac distance matrix Clusters I and II have a significantly distinct microbiota composition (PERMANOVA adjusted for PY, p = 0.001). Grey arrows represent the 3 most relevant ASVs for cluster segregation. One ASV of *Prevotella melaninogenica (d0b698c7298bf04110a6d2f220879bfb)* is the major contributor for segregation of Cluster II, while one ASV of *Haemophilus parainfluenzae (e27680d4009f98f30248d823bc17fb8e)* and another for *Streptococcus sp. (a5189f77a2cfeab3bc1602ff5c8ac3e9) c*ontribute for segregation of Cluster I. **C** The microbiota of Cluster I is less diverse than microbiota of Cluster 2 (Mann–Whitney U-test, U = 271, *p* = 0.008). *p < 0.05, **p < 0.01, ***p < 0.001, ****p < 0.0001

In an effort to understand to what extent oral bacteria is able to stratify COPD we performed a clustering analysis using the salivary microbial composition of patients. This analysis separated 90% of the individuals in two well supported clusters (“Cluster I” bootstrap node support (bns) = 74% and “Cluster II”, bsn = 84%; Fig. 2) which significantly differed in disease severity.

Cluster I aggregated all subjects with a history of recent severe exacerbation leading to hospital admission (Chi-square test, Z = 5.01, *p* = 0.025)) and 71% of the GOLD D (Chi-square test, Z = 1.98, *p* = 0.048). Two thirds of those under long term oxygen therapy or with heavier smoking history were also allocated to Cluster I. No other clinical parameters showed significant differences between the two clusters (Additional file 2: Table S2).

Oral bacteria composition was significantly different between the two clusters (PERMANOVA adjusted for PY, *P* = 0.001. Figure 2b.). Cluster I was enriched in patients dominated by Firmicutes or Proteobacteria, whereas cluster II was mainly represented by patients dominated by Bacteroidetes.

Oral bacteria diversity among patients (alpha diversity) was lower in Cluster I than in Cluster II (Fig. 2c. Mann–Whitney U-test, U = 271, *p* = 0.008). Similar differences were observed after adjusting for PY (ANOVA, F-value = 5.6, p = 0.006).

Regarding differentially abundant bacteria, both LEfSe and ANCOM distinguished Cluster I as particularly enriched in *Streptococcus* (Firmicutes) and detected *Prevotella* and *Alloprevotella* as responsible for the overabundance of Bacteroidetes in Cluster II (see Additional file 2: Fig. S2 for the complete list of OTUs detected by LEfSe). Both methods further detected a significant enrichment of Dialister (Firmicutes) in Cluster II.

Logistic regression analyses were performed to quantify the risk afforded by the prevalence of Firmicutes, Proteobacteria and Bacteroidetes in oral bacteria of people with COPD belonging to the two clusters. Furthermore, since three ASVs belonging to each of these phyla (*Prevotella melaninogenica* (Bacteroidetes), *Haemophilus parainfluenzae* (Proteobacteria) and *Streptococcus sp.* (Firmicutes)) were the main responsible for cluster segregation (Fig. 2b), the predictive power of their frequency was also inspected.

**Fig. 3:**
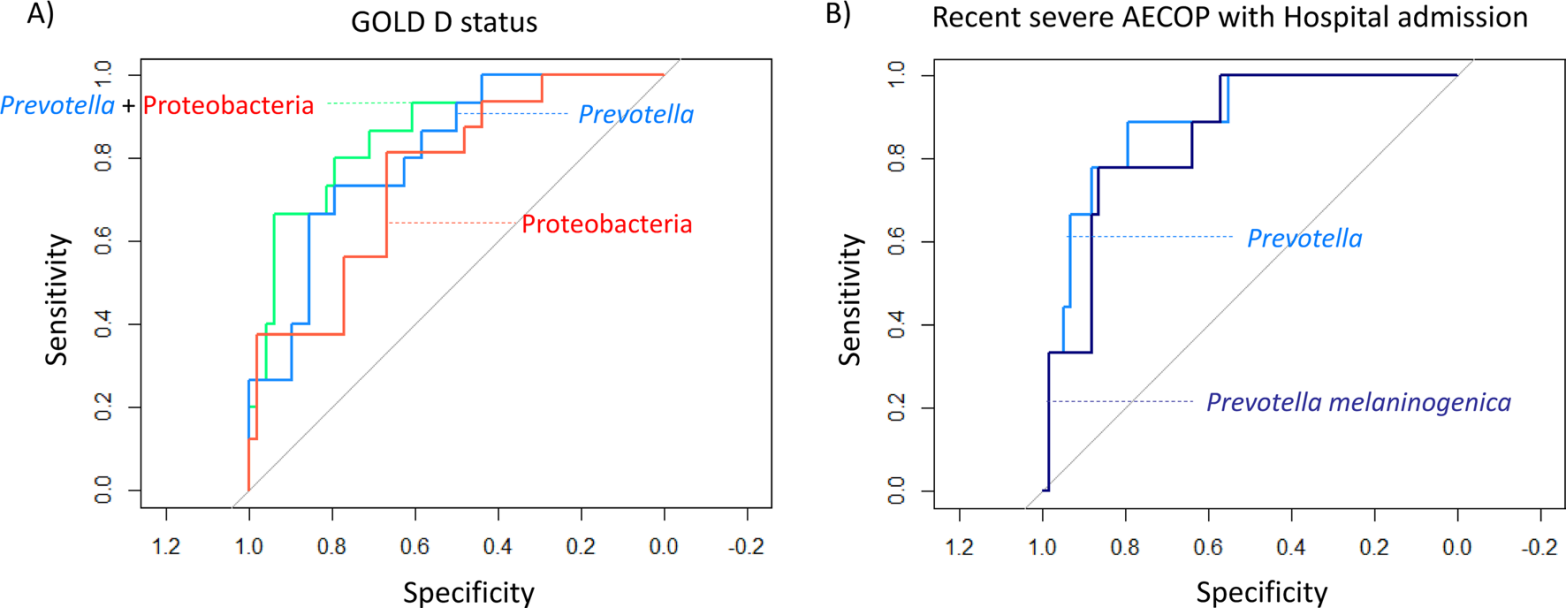
ROC analyses of GOLD D status prediction and recent severe exacerbation status prediction based on logistic regression models adjusted for Pack-years. **A** ROC curves of GOLD D status prediction based on relative frequency of *Prevotella*, Proteobacteria and *Prevotella* + Proteobacteria. The blue curve represents the prediction based on *Prevotella* relative frequency (AUC = 81%), the red curve represents the prediction based on Proteobacteria relative frequency (AUC = 75%) and the green curve represents the prediction based on the two regressors model (AUC = 87%). **B** ROC curves of recent severe exacerbation prediction based on relative frequency of *Prevotella genus* and *Prevotella melaninogenica* ASV *(d0b698c7298bf04110a6d2f220879bfb)*. Light blue curve represents the prediction made with *Prevotella* genus relative frequency (AUC = 89%), dark blue curve represents the prediction made with *Prevotella melaninogenica* ASV relative frequency (AUC = 86%)

The combined frequency of *Prevotella* (Bacteroidetes) and Proteobacteria was found to be the best predictor of being GOLD D, (AUC = 87%), Additional file 2: Table S3 and Fig. 3a), i.e., patients with lower frequency of *Prevotella* and higher frequency of Proteobacteria were more likely to be severe.

Moreover, the odds ratio (OR) of 0.44 suggests a protective effect for increasing frequencies of *Prevotella*, while the OR of 2.83 suggests a risk effect for increasing frequencies of Proteobacteria.

*Prevotella* was the best predictor of recent severe exacerbation (leading to hospital admission) (AUC = 89%), which translated into a significantly higher risk for patients with low frequencies of this genus. The OR of 0.58, corroborated the protective effect (Additional file 2: Table S3 and Fig. 3b). Additionally, the frequency of *Prevotella melaninogenica* alone was also a good predictor for recent severe exacerbation (AUC = 86%) (Additional file 2: Table S3 and Fig. 3b), similarly the OR of 0.63 suggests a protective effect for higher frequencies of this ASV.

No significant associations were found considering Bacteroidetes, *Haemophilus*, *Haemophilus parainfluenzae*, Firmicutes, *Streptococcus* or *Streptococcus sp.* relative frequencies.

## Discussion

Overall, our data suggests that the presence of an abundant Bacteroidetes community (dominated by commensal *Prevotella* species) in patients’ oral bacteria could have a protective effect towards severe COPD exacerbations.

Strikingly, we observed the separation of patients with recent history of severe exacerbation from all others based on oral bacteria, with low frequencies of *Prevotella* being the signature of this event. Possible mechanisms underlying this effect include the *Prevotella*-induced reduction of lung epithelial cell permeability (by modulating the expression of tight junction proteins) [42] or *Prevotella*-induced microbiota stabilization and resistance to pathobionts colonization [43].

The same phyla dominating the profile of patients with a history of recent severe exacerbation have been previously reported to be enriched in patients with higher predisposition for exacerbations [44, 45].

The depletion of *Prevotella* and the increased frequencies of *Haemophilus* and *Streptococcus* in patients with history of recent severe exacerbations corroborate the vicious cycle hypothesis [46]. According to this theory, the prolonged exposure to tobacco smoke (cluster I aggregated a greater proportion of heavier smokers) induces inflammation in the lung with increased levels of oxidative stress, protease imbalance and mucus hypersecretion. Consequently, an exacerbated, but not efficient, innate immune response allows for facultative anaerobes, e.g. *Haemophilus* which are better fitted, to persist and proliferate in the lung, enhancing further derangements in innate immunity mechanisms, and possibly triggering COPD exacerbations. Even after smoking cessation, the repetitive cycles of microbiota dysbiosis, together with impaired immune response cause irreversible structural modifications in the small airways and alveoli contributing for COPD progression.

Conversely, less severe patients displayed an overabundance of *Prevotella,* characteristic of healthy subjects [47].

*Prevotella* is the most abundant genus in the respiratory tract of healthy individuals [48] with some species having inflammatory properties [49], but most members likely being commensals. Among these, in our study, *P. melaninogenica* was depleted in the most severe grades of the disease and showed a potential protective effect against severe exacerbations. Interestingly the same species has been previously reported to have a protective role in vitro. By co-cultivating *P. melaninogenica* and *H. influenza,* Larsen et al. [50] demonstrated that *P. melaninogenica* modulated the in vitro inflammatory response of human dendritic cells induced by *H. influenzae*.

The characterization of oral bacteria of people with COPD and healthy individuals broadly corroborated the main differences previously observed in the lower respiratory tract (e.g. sputum or BAL) [4, 7, 51]: an expansion of Proteobacteria in patients and a Firmicutes and Bacteroidetes enrichment in healthy. Moreover, the overrepresentation of *Granullicatella* in moderate patients and *Haemophilus* in severe patients in our study, matches the observations in sputum of people with COPD [4, 7, 51]. Loss of microbial diversity, considered a signature of dysbiosis [52] due to its importance for microbiota stability [53], was also present in people with COPD. This is compatible with less complex airway microbiotas having a lower resistance to colonization by pathobionts [54], such as *Haemophilus,* frequently implicated in COPD exacerbations.

The microbiota characterization of clinically defined groups lies on the assumption of a correspondence between clinical categories and microbial profiles. Nevertheless, since COPD is a complex disease, enforcing such a correspondence might obscure the relationship between microbiota and the disease.

To explore the potential of the microbiota to stratify the disease, we performed an unsupervised clustering analysis of the diseased population. Interestingly, this analysis separated the individuals in two groups displaying different severities but showed only a week correspondence with the obstruction level, which is the criterion to diagnose the disease.

Some limitations of our study need to be acknowledged. First, although innovative and with great potential as a prognostic biomarker for COPD, the salivary bacterial community is susceptible to be influenced by oral health and smoking habits. For example, periodontitis is likely to influence the salivary microbiota [55] and has been previously associated with COPD [56]. Nevertheless, the bacterial groups found by two recent publications distinguishing the salivary microbiota of healthy individuals from people with periodontitis [55] or from people with periodontitis concomitant with COPD [57], were not coincident with the ones found by our study. Though we cannot discard the influence of this oral disease in our work it does not seem to be a major factor to differentiate patients from healthy.

Second, we were not able to evaluate the prospective effects of high frequencies of *Prevotella* in terms of preventing severe COPD exacerbations due to the cross-sectional study design. Further studies should explore these effects prospectively.

Third, we acknowledged that the use of saliva is still exploratory and that external validation of our findings in a multicentre trial and with larger cohorts is needed to assess its robustness, especially in a disease as heterogeneous as COPD.

In conclusion, based on the analysis of people with COPD and healthy individuals, our data suggests an association between oral bacteria and COPD, particularly in terms of severe exacerbations. It further shows that even in stability it is possible to identify the dysbiotic microbial signatures associated with severe COPD exacerbations.

Additionally, our results suggest that unsupervised analyses of oral bacteria may provide a more useful insight into its relationship with the disease potentially enabling practical applications such as risk assessment and patient counselling.

**Table 1:** Sociodemographic, anthropometric and clinical characteristics of participants included in the study

Characteristics	COPD (n = 70)	HEALTHY (n = 58)	*p-*value
Age (years), mean ± SD	67.9 ± 8.7	67.0 ± 8.2	0.7
Male sex, n (%)	60 (86%)	42 (84%)	0.8
BMI (kg/m^2^), mean ± SD	25.5 ± 3.5	27.6 ± 3.8	0.001
Pack-years, mean ± SD	42.2 ± 45.3	8 ± 21.0	< 0.0001
CCI, mean ± SD	3.7 ± 1.3	2.0 ± 1.0	< 0.0001
Medication for COPD, n (%)	70 (100%)	0 (0%)	
Smoking status, n (%)			
Current smoker	7 (10%)	2 (4%)	< 0.0001
Former smoker	49 (70%)	10 (20%)	
Never smoker	14 (20%)	38 (76%)	
GOLD Grade, n (%)			
1	7 (10%)	n.a	
2	25 (36%)	n.a	
3	26 (37%)	n.a	
4	12 (17%)	n.a	
GOLD Group, n (%)			
A	12 (17%)	n.a	
B	32 (46%)	n.a	
C	5 (7%)	n.a	
D	21 (30%)	n.a	
Long-term oxygen dependence, n (%)	11 (16%)	0 (0)	
SpO_2_, mean ± SD (%)	94.4 ± 1.9	96.7 ± 1.7	< 0.0001
FEV_1_ (L)	1.3 ± 0.4	2.8 ± 0.6	< 0.0001
FEV_1_pp, mean ± SD	48.0 ± 16.4	103.0 ± 16.7	< 0.0001
FVC (L)	2.7 ± 0.6	3.4 ± 0.7	< 0.0001
Ratio FEV_1_FVC	48.7 ± 12.1	83.8 ± 8.7	< 0.0001
Number of exacerbations in the year before enrolment, n (%)			
0–1	49 (70%)	n.a	
≥ 2 or 1 with hospital admission	21 (30%)	n.a	
Hospital admissions due to COPD, in the year before enrolment, n (%)			
0	60 (86%)	n.a	
1	10 (14%)	n.a	

Comparisons between people with COPD and Healthy controls were conducted with unpaired t-test with Welch’s correction, Mann–Whitney U-test and Fisher’s exact test. n (%): number of individuals in each group plus the corresponding percentage. mean±SD: mean±standard deviation. CCI: Charlson Comorbidity Index; BMI: Body Mass Index; GOLD Grade: 3—Severe; 4—Very Severe; GOLD Group: A—Less symptoms and low risk of exacerbations; B—More symptoms and low risk of exacerbations; C– Less symptoms and high risk of exacerbations; D—More symptoms and high risk of exacerbations; FEV1pp: forced expiratory volume in 1 second percentage of predicted; SpO2: peripheral capillary oxygen saturation. Comparisons between patients with COPD and Healthy controls were conducted with unpaired t-test with Welch’s correction, Mann-Whitney U-test and Fisher’s exact test

## Supplementary Information

**Additional file 1: Table S1.** Sociodemographic, anthropometric and clinical database of study participants.

**Additional file 2: Supplementary Methods. Table S2.** Summary of clinical parameters distribution across Clusters I and II. Comparisons between clusters were conducted with Mann-Whitney U-test and chi-square test. **Table S3**. Summary table of significant logistic regression models established for both GOLD D and hospital admission, adjusted for Pack-years. Coefficients were represented in model equations. **Figure S1**. Salivary bacteria composition is different between patients with COPD and healthy controls. A) Bar-plot representing the differentially abundant genera between moderate patients with COPD and healthy controls inferred by LEfSe at a significance cut-off of 3. B) Bar-plot representing the differentially abundant genera between moderate patients with COPD and healthy controls inferred by LEfSe at a significance cut-off of 3. C) Bar-plot representing the differentially abundant genera between severe patients with COPD and healthy controls inferred by LEfSe at a significance cut-off of 3. A), B) and C) Differentially abundant OTUs inferred by ANCOM at 0.7 significance cut-off are underlined. **Figure S2**. Salivary bacteria composition is different between the two clusters. Bar-plot representing the differentially abundant genera between cluster I and cluster II inferred by LEfSe at a significance cut-off of 3. Differentially abundant OTUs inferred by ANCOM at 0.7 significance cut-off are underlined. 

## Data Availability

The dataset supporting the conclusions of this article is included within the article (and its additional file(s)). Furthermore, raw sequencing data is deposited in National Centre for Biotechnology Information's (NCBI) Sequence Read Archive (SRA) (BioProject PRJNA800368).
