# Correction: Saliva as a non‑invasive specimen for COPD assessment

**DOI:** 10.1186/s12931-022-02249-6

**Published:** 2022-12-21

**Authors:** Sara Melo‑Dias, Carla Valente, Lília Andrade, Alda Marques, Ana Sousa

**Affiliations:** 1 Department of Medical Sciences, University of Aveiro, Aveiro, Portugal; 2 Lab3R‑Respiratory Research and Rehabilitation Laboratory, School of Health Sciences (ESSUA), University of Aveiro, Aveiro, Portugal; 3 Institute of Biomedicine (iBiMED), University of Aveiro, 3810‑193 Aveiro, Portugal; 4 Department of Pulmonology, Hospital Center of Baixo Vouga, Aveiro, Portugal

## Correction:  Respiratory Research (2022) 23:16 https://doi.org/10.1186/s12931-022-01935-9

Following the publication of the original article [1], it was noted that the Supplementary file 2 has been processed incorrectly.

The correct Additional file 2—supplementary methods been updated and included in this correction.

The original article has been corrected.

### Supplementary Information

**Additional file 2.** **Supplementary Methods. Supplementary table S2.** Summary of clinical parameters distribution across Clusters I and II. Comparisons between clusters were conducted with Mann-Whitney U-test and chi-square test. **Supplementary table S3.** Summary table of significant logistic regression models established for both GOLD D and hospital admission, adjusted for Pack-years. Coefficients were represented in model equations. **Supplementary figure S1.** Salivary bacteria composition is different between patients with COPD and healthy controls. **A**) Bar-plot representing the differentially abundant genera between moderate patients with COPD and healthy controls inferred by LEfSe at a significance cut-off of 3. **B**) Bar-plot representing the differentially abundant genera between moderate patients with COPD and healthy controls inferred by LEfSe at a significance cut-off of 3. C) Bar-plot representing the differentially abundant genera between severe patients with COPD and healthy controls inferred by LEfSe at a significance cut-off of 3. **A**), **B**) and **C**) the differential OTUs inferred by ANCOM at 0.7 significance cut-off are represented in underlined. **Supplementary figure S2.** Salivary bacteria composition is different between the two clusters. Bar-plot representing the differentially abundant genera between cluster 1 and cluster 2 inferred by LEfSe at a significance cut-off of 3. The differential OTUs inferred by ANCOM are represented in underlined at 0.7 signofciance cut-off.
