# Supplementary material for: Saliva as a non-invasive specimen for COPD assessment
Source: Respir Res. 2022 Jan 29;23:16. doi: 10.1186/s12931-022-01935-9 (PMC8800366; doi:10.1186/s12931-022-01935-9)
Supplement: Supplementary file 2 — Additional file 2: Supplementary Methods. Table S2. Summary of clinical parameters distribution across Clusters I and II. Comparisons between clusters were conducted with Mann-Whitney U-test and chi-square test. Table S3. Summary table of significant logistic regression models established for both GOLD D and hospital admission, adjusted for Pack-years. Coefficients were represented in model equations. Figure S1. Salivary bacteria composition is different between patients with COPD and healthy controls. A) Bar-plot representing the differentially abundant genera between moderate patients with COPD and healthy controls inferred by LEfSe at a significance cut-off of 3. B) Bar-plot representing the differentially abundant genera between moderate patients with COPD and healthy controls inferred by LEfSe at a significance cut-off of 3. C) Bar-plot representing the differentially abundant genera between severe patients with COPD and healthy controls inferred by LEfSe at a significance cut-off of 3. A), B) and C) Differentially abundant OTUs inferred by ANCOM at 0.7 significance cut-off are underlined. Figure S2. Salivary bacteria composition is different between the two clusters. Bar-plot representing the differentially abundant genera between cluster I and cluster II inferred by LEfSe at a significance cut-off of 3. Differentially abundant OTUs inferred by ANCOM at 0.7 significance cut-off are underlined. [file 12931_2022_1935_MOESM2_ESM.docx]

# SALIVA AS A NON-INVASIVE SPECIMEN FOR COPD ASSESSEMENT

Sara Melo-Dias^1,2,3^, Msc, Carla Valente^5^, MD, Lília Andrade^5^, MD, Alda Marques^2,3,^, PhDǂ and Ana Sousa^1,3^, PhDǂ

**SUPPLEMENTARY MATERIAL**

# Methods

A cross-sectional study was conducted. Ethical approvals were obtained from Administração Regional de Saúde Centro (64/2016) and from Centro Hospitalar do Baixo Vouga (08-03-17). Written informed consent was obtained from all participants.

## Subjects and sample collection

Patients were eligible if diagnosed with COPD according to the Global Initiative for Chronic Obstructive Lung Disease (GOLD) criteria [1] and were stable with no acute exacerbations in the month prior to enrolment. Exclusion criteria were presence of severe cardiac, musculoskeletal, or neuromuscular diseases, cognitive impairment, active neoplasia or immune diseases. Healthy-individuals were age- and sex-matched to patients and had similar inclusion and exclusion criteria except for the absence of any respiratory disease. Sociodemographic, anthropometric, clinical data and saliva samples (passive drool method) were collected using a structured protocol [2].

Sociodemographic (age, sex, educational level), anthropometric (weight and height to compute body mass index), clinical (smoking habits, number of exacerbations and hospitalizations in the past year, medication used, long-term oxygen, comorbidities - Charlson Comorbidity Index [3], level of airway obstruction-spirometry (FEV_1_, FVC, FEV_1_pp) [1], medication including long term oxygen therapy, self-perceived dyspnoea during activities - modified Medical Research Council Questionnaire [4], impact of the disease – COPD Assessment Test (CAT) [5] data and saliva samples, passive drool method, were collected with a structured protocol adapted from the team published work [2]. GOLD grades were defined according to FEV1 percentage predicted for each individual. GOLD groups were defined combining the number of exacerbations and hospital admissions of each patient in the year before enrolment with their CAT scores. Before saliva sample collection the patient was advised to drink a glass of water (especially if he had recently drunk coffee or citrus juice) and to provide 3-4 mL of saliva using a labelled sample collection cup. Subsequently, the sample was transported in a cooler to the lab as quickly as possible and preserved at -80ºC until DNA extraction.

## DNA extraction

Prior to DNA extraction, samples were thawed at room temperature and centrifuged at 10,000xg for 10 minutes. Supernatants were discarded and DNA extraction from cell pellets followed QIAamp DNA Mini Kit (Qiagen, Hilden, Germany), protocol with minor modifications: initial sample volume was set to 400µL and the volumes of buffers and Qiagen protease were adjusted. Elution volume was reduced to a quarter of the recommended. Thirteen negative controls where saliva was replaced by phosphate-buffered saline were performed in order to control for background bacterial contamination. Quality and quantity of the extracted DNA was assessed in Denovix DS-11 spectrophotometer, with OD260/280 and OD260/230 ratios.

## 16S rRNA gene amplification and sequencing


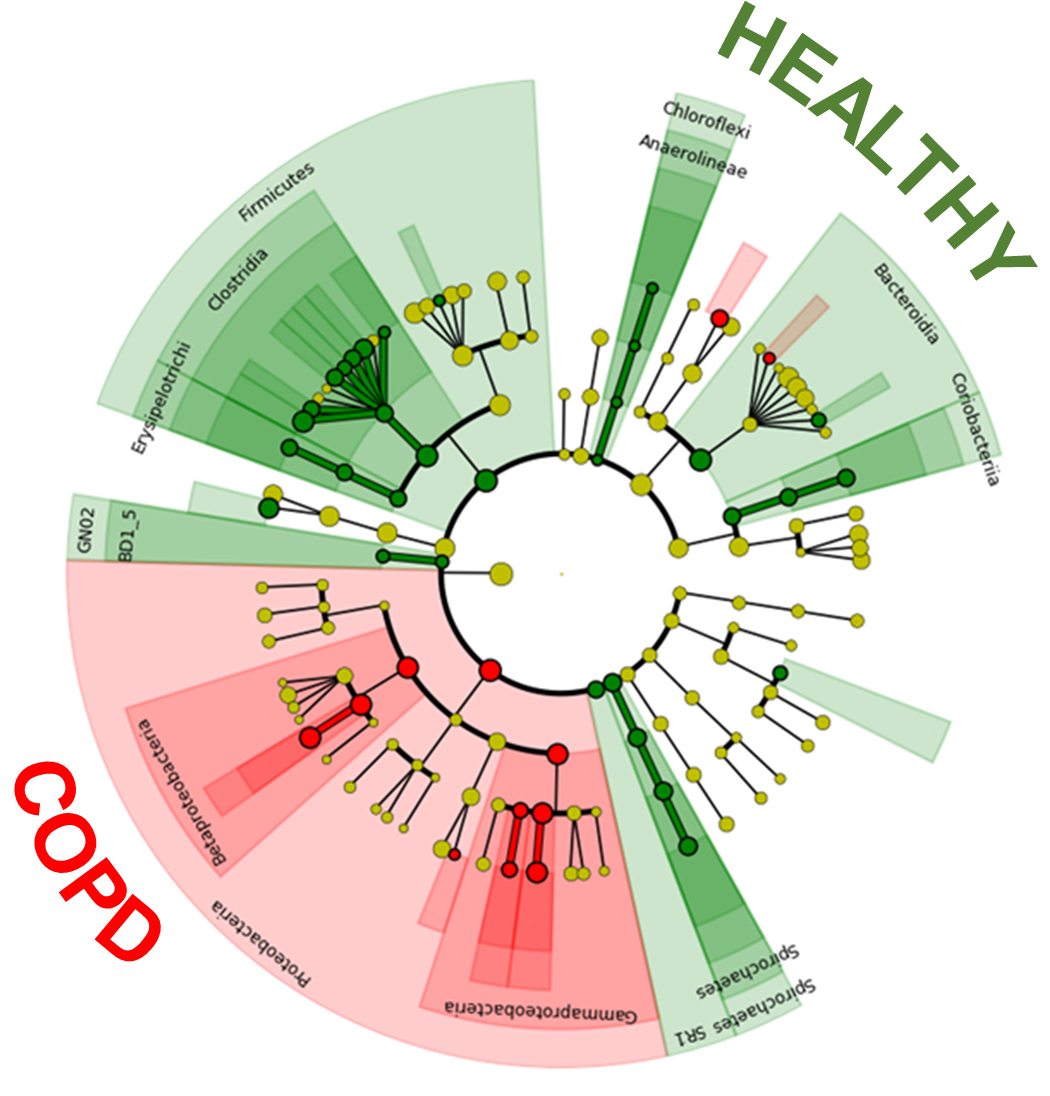
16S rRNA gene amplification and sequencing was carried out at the Gene Expression Unit from Instituto Gulbenkian de Ciência following the implemented protocol. Briefly, for each sample, the hypervariable V4 region of 16S rRNA gene was amplified, using universal pair of primers F515 (5′-CACGGTCGKCGGCGCCATT-3′) / R806 (5′-GGACTACHVGGGTWTCTAAT-3′). Samples were then pair-end-sequenced on an Illumina MiSeq Benchtop Sequencer, following Illumina recommendations.

## Microbiota and statistical analyses

*Sample characterisation*

Descriptive statistics was used to describe the sample, normality of data was assessed with Shapiro-Wilk normality test, ensuring the assumptions of parametric statistics approach. Comparisons between patients with COPD and Healthy controls were conducted with unpaired t-test with Welch’s correction, when quantitative data followed normal distribution, Mann-Whitney U-test, when quantitative data violated the assumptions of parametric tests (assessed with Kolmogorov-Smirnov test) and Chi-square test, when qualitative data was considered (statistical analyses were performed in *GraphPad Prism 8* [6] and *R* software (v.3.6.0) [7]).

*Analysis of Illumina paired-end reads*

QIIME2 2020.8 [8,9] was used to perform microbiota analyses. Demultiplexed 16s paired-end sequences were imported and q2-vsearch plugin [10] was applied to join forward and reverse reads. Quality control was assessed via q-score base filtering (joined reads with a Phred score bellow 30 were remove), chimera removing and 16S-denoising with Deblur [11]. Next, to exclude bacterial contaminations, we used the *DECONTAM* package [12,13] from *R* software [7] which allowed us to identify 171 contaminant ASVs by evaluating the prevalence (presence/absence across samples of each DNA extraction batch) of each ASV in the saliva samples compared to the prevalence in the respective negative controls. These ASVs together with ASVs from *mitochondria*, *chloroplasts* and *cyanobacteria*, and the negative control samples were removed from the dataset prior to conducting subsequent analyses. Results from previous steps were summarized in a feature table. Q2-phylogeny [14] plugin from QIIME2 [8,9] was next employed to produce a MAFFT alignment [15] of ASVs which was consequently used to construct a rooted phylogeny with FastTree2 [16] for subsequent applications.

Taxonomy assignment of ASVs was performed with q2-feature-classifier plugin [17,18], through classify-sklearn method with pre-trained Naïve Bayes classifier against 99%-eHOMD_v15.1 reference sequences [19] (sequences trimmed to only include 250bp of V4 region, bound by the F515/R806 primer pair).

All the subsequent analyses, excepting the differential abundance, were performed with data upon ASVs. Differential abundance analyses were done with data upon OTUs at taxonomic level 6.

*Diversity analyses*

Alpha- and beta-diversities were estimated q2-diversity plugin [20] after rarefaction of samples (subsample without replacement) to 4000 sequences per sample as implemented in QIIME2 [8,9]. Spatial dissimilarities between bacterial communities of different groups were assessed with principal coordinate analyses (PCoA) and biplots based on Weighted Unifrac distance. Mann-Whitney U-test and Kruskal-Wallis with Dunn’s correction were employed to compare alpha diversity among groups (statistical analyses were performed in *GraphPad Prism 8* [6] and *R stats* package [21] from *R* software [7]). Additionally, the effect of disease state (COPD vs healthy) on alpha diversity indexes was adjusted for pack-years (PY) using a Linear Regression Model (*R stats* package [21] from *R* software [7]). Differences in beta-diversity between groups were quantified by Permutational multivariate analysis of variance (PERMANOVA) [22] was conducted with *adonis2* funcation [23] (*vegan* package [24] from *R* software [7]) adjusted for pack-years (PY). For all statistical analyses a p-value of ≤0.05, corrected for multiple testing whenever necessary, was considered statistically significant.

*Differential abundance analyses of OTUs*

Analysis of composition of microbiomes (ANCOM) [25] and Linear discriminant effect size (LEfSe) analysis [26] were performed to identify differentially abundant OTUs between clusters. LEfSe is one algorithm for high-dimensional biomarker discovery that uses linear discriminant analysis to estimate the effect size of each taxon [26] and does not account for the compositional nature of the microbiota, and ANCOM uses a log ratio analysis to make point estimates of the variance and mean [25], taking into consideration the compositional nature of the data. These analyses were conducted with the feature table collapsed at genus taxonomic level (L6). LEfSe was performed in the online version [27] with an Linear Discriminant Analysis score of 3 for significance. ANCOM was performed in *R* software with *ANCOM 2.0* script [28] with taxa-wise multiple correction and a W cut-off of significance of 0.7. (both recommended by the developer, based on simulation data).

*Clustering analysis*

A hierarchical clustering analysis of the microbiota was performed with beta rarefaction script [29] available in q2-diversity plugin [20] from QIIME2 [8,9] at minimum read depth (4000 reads). This built 5000 rarefied feature tables, computed Weighted Unifrac and generated a consensus tree with neighbour joining support of two nodes. Mann-Whitney U-test and Chi-square test were used describe the differences of clinical features among clusters (statistical analyses were performed in *GraphPad Prism 8* [6] and *R stats* package [21] from *R* software [7]).

*Binary logistic regression models ROC analyses*

To further explore the relation between the more relevant OTUs/ASVs and clinical features in the context of the clustering analysis, logistic regression models and risk assessment analyses were performed.

Binary logistic regression models, adjusted for pack-years, were established using glm[30] (link=logit) function of *R stats* package [21] *R* software [7]. Log_2_ transformed relative frequencies of most discriminant ASVs between clusters and the frequencies of the genus and phylum form each ASV were explored as possible regressors for having had an hospital admission as consequence of a severe COPD exacerbation and for being a GOLD D patient (most severe in terms of GOLD groups). Residual analyses were performed with a threshold cut-off of 1.96 for outliers’ removal. Akaike information criterion (AIC) with confidence intervals (CI) of 95% was used to assess the models' quality (*aod* package [31] from *R* software [7]). Models without outliers were preferred whenever the AIC was lower than the one from the correspondent model with outliers and when the number of outliers identified was appropriate considering the sample size.  The quality of the fit of models was estimated, only for models with significant regressors, with Likelihood ratio test (*lmtest* package [32] from *R* software [7]), deviance, Nagelkerke's pseudo R2 [33] (*DescTools* package [34] from *R* software [7]) and Hosmer-Lemeshow test [35] (*ResourceSelection* package [36] from *R* software [7]). The exponentiated coefficients, which correspond to the Odds ratio of each regressor, were calculated for each significant model. Receiver operating characteristic curves (ROC) and the respective area under the curve (AUC) for each significant model were also calculated (*pROC* package [37] from *R* software [7]).

# References

1. GOLD - Global Strategy for Diagnosis, Management, and prevention of chronic obstructive pulmonary disease 2020 report. 2020. 1–141.

2. Marques A, Jácome C, Rebelo P, Paixão C, Oliveira A, Cruz J, et al. Improving access to community-based pulmonary rehabilitation: 3R protocol for real-world settings with cost-benefit analysis. BMC Public Health. 2019;19:676.

3. Charlson ME, Pompei P, Ales KL, MacKenzie CR. A new method of classifying prognostic comorbidity in longitudinal studies: development and validation. J Chronic Dis. 1987;40:373–83.

4. Bestall J, Paul E, Garrod R, Garnham R, Jones P, Wedzicha J. Usefulness of the Medical Research Council (MRC) dyspnoea scale as a measure of disability in patients with chronic obstructive pulmonary disease. Thorax. 1999;54:581–6.

5. Jones PW, Harding G, Berry P, Wiklund I, Chen W-H, Kline Leidy N. Development and first validation of the COPD Assessment Test. European Respiratory Journal. 2009;34:648–54.

6. Prism - GraphPad [Internet]. [cited 2022 Jan 12]. Available from: https://www.graphpad.com/scientific-software/prism/

7. R: The R Project for Statistical Computing [Internet]. [cited 2022 Jan 12]. Available from: https://www.r-project.org/

8. Bolyen E, Rideout JR, Dillon MR, Bokulich NA, Abnet CC, Al-Ghalith GA, et al. Reproducible, interactive, scalable and extensible microbiome data science using QIIME 2. Nat Biotechnol. 2019;37:852–7.

9. QIIME 2 [Internet]. [cited 2022 Jan 12]. Available from: https://qiime2.org/

10. vsearch — QIIME 2 2020.8.0 documentation [Internet]. [cited 2021 Dec 30]. Available from: https://docs.qiime2.org/2020.8/plugins/available/vsearch/

11. deblur — QIIME 2 2020.8.0 documentation [Internet]. [cited 2021 Dec 30]. Available from: https://docs.qiime2.org/2020.8/plugins/available/deblur/

12. Callahan B, Davis NM, Ernst FGM. decontam: Identify Contaminants in Marker-gene and Metagenomics Sequencing Data [Internet]. Bioconductor version: Release (3.14); 2021 [cited 2021 Dec 30]. Available from: https://bioconductor.org/packages/decontam/

13. Callahan B. benjjneb/decontam [Internet]. 2021 [cited 2022 Jan 12]. Available from: https://github.com/benjjneb/decontam

14. Phylogenetic inference with q2-phylogeny — QIIME 2 2021.11.0 documentation [Internet]. [cited 2021 Dec 30]. Available from: https://docs.qiime2.org/2021.11/tutorials/phylogeny/?highlight=phylogeny

15. Katoh K, Misawa K, Kuma K, Miyata T. MAFFT: a novel method for rapid multiple sequence alignment based on fast Fourier transform. Nucleic Acids Res. 2002;30:3059–66.

16. FastTree 2 – Approximately Maximum-Likelihood Trees for Large Alignments [Internet]. [cited 2019 Jul 23]. Available from: https://journals.plos.org/plosone/article?id=10.1371/journal.pone.0009490

17. Bokulich NA, Kaehler BD, Rideout JR, Dillon M, Bolyen E, Knight R, et al. Optimizing taxonomic classification of marker-gene amplicon sequences with QIIME 2’s q2-feature-classifier plugin. Microbiome. 2018;6:90.

18. QIIME 2 Library [Internet]. [cited 2021 Dec 30]. Available from: https://library.qiime2.org/plugins/q2-feature-classifier/3/

19. F. Escapa I, Huang Y, Chen T, Lin M, Kokaras A, Dewhirst FE, et al. Construction of habitat-specific training sets to achieve species-level assignment in 16S rRNA gene datasets. Microbiome. 2020;8:65.

20. diversity — QIIME 2 2020.8.0 documentation [Internet]. [cited 2021 Dec 30]. Available from: https://docs.qiime2.org/2020.8/plugins/available/diversity/

21. R: The R Stats Package [Internet]. [cited 2022 Jan 12]. Available from: https://stat.ethz.ch/R-manual/R-devel/library/stats/html/00Index.html

22. Anderson MJ. A new method for non-parametric multivariate analysis of variance. Austral Ecology. 2001;26:32–46.

23. adonis function - RDocumentation [Internet]. [cited 2021 Sep 15]. Available from: https://www.rdocumentation.org/packages/vegan/versions/2.4-2/topics/adonis

24. vegan-package: Community Ecology Package: Ordination, Diversity and... in vegan: Community Ecology Package [Internet]. [cited 2022 Jan 12]. Available from: https://rdrr.io/cran/vegan/man/vegan-package.html

25. Mandal S, Van Treuren W, White RA, Eggesbø M, Knight R, Peddada SD. Analysis of composition of microbiomes: a novel method for studying microbial composition. Microb Ecol Health Dis. 2015;26:27663.

26. Segata N, Izard J, Waldron L, Gevers D, Miropolsky L, Garrett WS, et al. Metagenomic biomarker discovery and explanation. Genome Biol. 2011;12:R60.

27. Huttenhower C. Galaxy / Hutlab - Harvard [Internet]. [cited 2019 Sep 19]. Available from: http://huttenhower.sph.harvard.edu/galaxy/

28. Mandal, Siddhartha. Research - Dr. Siddhartha Mandal: ANCOM 2.0 - updated code [Internet]. [cited 2019 Sep 19]. Available from: https://sites.google.com/site/siddharthamandal1985/research

29. beta-rarefaction: Beta diversity rarefaction — QIIME 2 2020.8.0 documentation [Internet]. [cited 2021 Dec 30]. Available from: https://docs.qiime2.org/2020.8/plugins/available/diversity/beta-rarefaction/?highlight=beta%20rarefaction

30. glm function - RDocumentation [Internet]. [cited 2021 Mar 29]. Available from: https://www.rdocumentation.org/packages/stats/versions/3.6.2/topics/glm

31. Lancelot ML and R. aod: Analysis of Overdispersed Data [Internet]. 2019 [cited 2022 Jan 12]. Available from: https://CRAN.R-project.org/package=aod

32. Hothorn T, Zeileis A, Farebrother (pan.f) RW, Cummins (pan.f) C, Millo G, Mitchell D. lmtest: Testing Linear Regression Models [Internet]. 2021 [cited 2022 Jan 12]. Available from: https://CRAN.R-project.org/package=lmtest

33. PseudoR2 function - RDocumentation [Internet]. [cited 2021 Mar 29]. Available from: https://www.rdocumentation.org/packages/DescTools/versions/0.99.40/topics/PseudoR2

34. Signorell A, Aho K, Alfons A, Anderegg N, Aragon T, Arachchige C, et al. DescTools: Tools for Descriptive Statistics [Internet]. 2021 [cited 2022 Jan 12]. Available from: https://CRAN.R-project.org/package=DescTools

35. hoslem.test function - RDocumentation [Internet]. [cited 2021 Mar 29]. Available from: https://www.rdocumentation.org/packages/ResourceSelection/versions/0.3-5/topics/hoslem.test

36. Lele SR, Keim JL, Solymos P. ResourceSelection: Resource Selection (Probability) Functions for Use-Availability Data [Internet]. 2019 [cited 2022 Jan 12]. Available from: https://CRAN.R-project.org/package=ResourceSelection

37. Robin X, Turck N, Hainard A, Tiberti N, Lisacek F, Sanchez J-C, et al. pROC: Display and Analyze ROC Curves [Internet]. 2021 [cited 2021 Mar 29]. Available from: https://CRAN.R-project.org/package=pROC

# Supplementary tables

**Supplementary table 1. Clinical database containing sociodemographic, anthropometric, and clinical characteristics of participants included in the study**

- Supplementary Excel file

**Supplementary table 2. Summary of clinical parameters distribution across Clusters I and II. Comparisons between clusters were conducted with Mann-Whitney U-test and chi-square test.**

| ***Characteristics*** | ***Cluster I (n=29)*** | ***Cluster II (n=31)*** | ***p-value*** |  |
| --- | --- | --- | --- | --- |
| ***Age (years), mean±SD*** | 70.1±7.9 | 65.3±8.5 | 0.08 |  |
| ***Pack-Years, mean±SD*** | 42.4±36.6 | 36.9±49.4 | 0.23 |  |
| ***CCI, mean±SD*** | 3.9±1.48 | 3.5±1.1 | 0.37 |  |
| ***GOLD Grade, n (%)*** | | | | |
| 1 | 5 (83%) | 1 (17%) | 0.16 |  |
| 2 | 11 (50%) | 11 (50%) | 1 |  |
| *3* | 8 (36%) | 14 (64%) | 0.25 |  |
| *4* | 5 (50%) | 5 (50%) | 1 |  |
| ***GOLD Group, n (%)*** | | | | |
| *A* | 5 (45%) | 6 (55%) | 1 |  |
| *B* | 11 (37%) | 19 (63%) | 0.07 |  |
| *C* | 3 (60%) | 2 (40%) | 0.93 |  |
| *D* | 10 (71%) | 4 (29%) | **0.048** |  |
| ***Long-term oxygen dependence, n (%)*** | 6 (67%) | 3 (33%) | 0.4 |  |
| ***CAT scores, mean±SD*** | 15.0±8.9 | 17.7±9.1 | 0.26 |  |
| ***SpO_2_, mean±SD (%)*** | 94.5±2.2 | 94.5±2.5 | 0.95 |  |
| ***FEV_1_pp, mean±SD*** | 51.0±21.0 | 45.0±17.0 | 0.31 |  |
| ***Number of exacerbations in the year before enrolment, n (%)*** | | | | |
| *0-1* | 20 | 24 | 0.81 |  |
| *≥2 or 1 with hospital admission* | 9 | 7 |  |  |
| ***Hospital admissions due to COPD, in the year before enrolment, n (%)*** | | | | |
| *0* | 23 (43%) | 31 (57%) | **0.025** |  |
| *1* | 6 (100%) | 0 (0) |  |  |
| *n (%): number of individuals in each group plus the corresponding percentage. mean±SD: mean±standard deviation. CCI: Charlson Comorbidity Index; BMI: Body Mass Index; GOLD Grade: 3– Severe; 4– Very Severe; GOLD Group: A– Less symptoms and low risk of exacerbations; B– More symptoms and low risk of exacerbations; C– Less symptoms and high risk of exacerbations; D– More symptoms and high risk of exacerbations; FEV1pp: forced expiratory volume in 1 second percentage of predicted; CAT: COPD assessment test; SpO2: peripheral capillary oxygen saturation. Comparisons between patients with COPD and Healthy controls were conducted with unpaired t-test with Welch’s correction, Mann-Whitney U-test and Fisher’s exact test.* | | | | |

**Supplementary table 3. Summary table of significant logistic regression models established for both GOLD D and hospital admission, adjusted for Pack-years. Coefficients were represented in model equations.**

|  |  |  | **Likelihood ratio test^▪^** | | |  |  |  |
| --- | --- | --- | --- | --- | --- | --- | --- | --- |
| **Model equation** | **Coefficients** | **p-value** | **χ2** | **df** | **p-value** | **AIC^▪▪^** | **OR (IC 95%)** | **AUC** |
| Predicted logit of (**GOLD D**) = -3.92+(-0.73)***log_2_(*Prevotella*)**+(0.006)***log_2_(Pack-Years)** | ***Prevotella*** | 0.003 | 14.9 | 2 | 0.0006 | 60.3 | 0.48 (0.27; 0.74) | 81% |
|  | **Pack-years** | 0.46 |  |  |  |  | 1 (0.99; 1.02) |  |
| Predicted logit of (**GOLD D**) = 0.88+(1.1)***log_2_(Proteobacteria)+**(0.01)***log_2_(Pack-Years)** | **Proteobacteria** | 0.007 | 12.5 | 2 | 0.002 | 65.5 | 3 (1.48; 7.64) | 75% |
|  | **Pack-years** | 0.16 |  |  |  |  | 1.01 (1; 1.02) |  |
| Predicted logit of (**GOLD D**) = -1.84+(1.04)***log_2_(Proteobacteria)**+(-0.83)***log_2_(*Prevotella)+***(0.002)***log_2_(Pack-Years)** | **Proteobacteria** | 0.02 | 22.02 | 3 | <0.0001 | 55.1 | 2.83 (1.34; 7.65) | 87% |
|  | ***Prevotella*** | 0.002 |  |  |  |  | 0.44 (0.23; 0.69) |  |
|  | **Pack-years** | 0.8 |  |  |  |  | 1 (0.98; 1.02) |  |
| Predicted logit of (**Hospital admission**) = -4.28+(-0.53)***log_2_(*Prevotella*)**+(0.01)***log_2_(Pack-Years)** | ***Prevotella*** | 0.02 | 10.3 | 2 | 0.006 | 48.6 | 0.58 (0.36; 0.86) | 89% |
|  | **Pack-years** | 0.16 |  |  |  |  | 1.01 (0.99; 1.02) |  |
| Predicted logit of (**Hospital admission**) = -4.54+(-0.46)***log_2_(d0b698c7298bf04110a6d2f220879bfb)**+(0.01)***log_2_(Pack-Years)** | **d0b698c7298bf04110a6d2f220879bfb** | 0.005 | 12.6 | 2 | 0.002 | 46.3 | 0.63 (0.43; 0.84) | 86% |
|  | **Pack-years** | 0.1 |  |  |  |  | 1.01 (0.99; 1.03) |  |
| ▪Comparison between the established model and null model for either being GOLD D or having had a recent severe exacerbation with hospital admission  ▪▪The AICs presented for each model were lower than the AICs of null models for either being GOLD D or having had a recent severe exacerbation with hospital admission | | | | | | | | |

# Supplementary figures


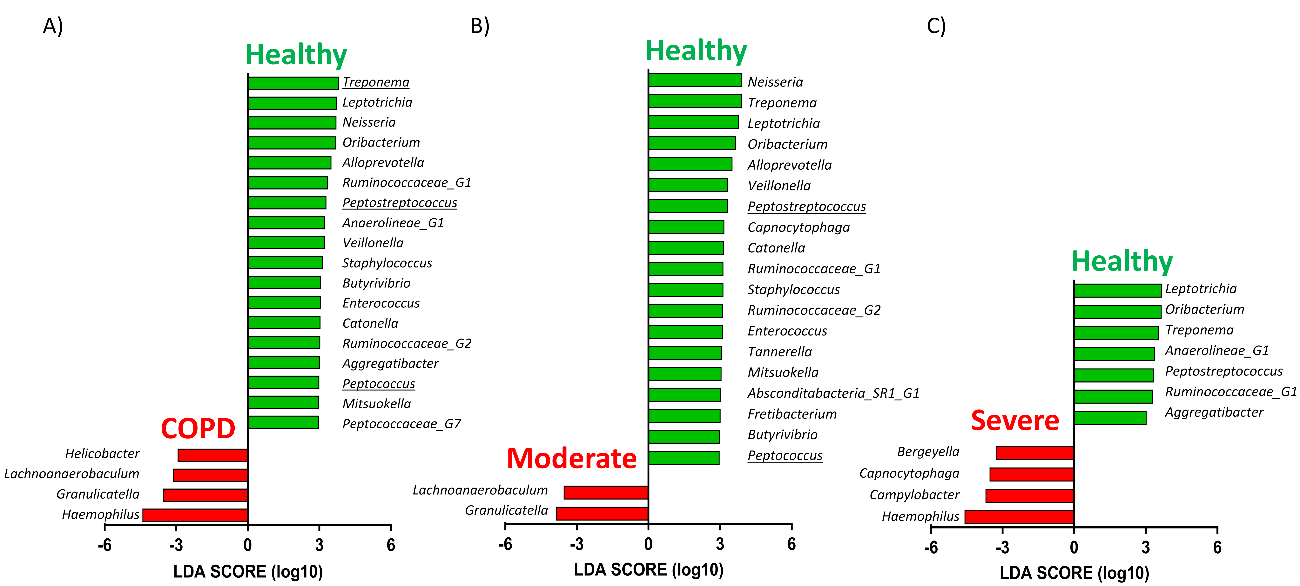


**Supplementary figure 1. Salivary microbiota composition is different between patients with COPD and healthy controls. A)** Bar-plot representing the differentially abundant genera between moderate patients with COPD and healthy controls inferred by LEfSe at a significance cut-off of 3. **B)** Bar-plot representing the differentially abundant genera between moderate patients with COPD and healthy controls inferred by LEfSe at a significance cut-off of 3. **C)** Bar-plot representing the differentially abundant genera between severe patients with COPD and healthy controls inferred by LEfSe at a significance cut-off of 3. **A), B)** and **C)** the differential OTUs inferred by ANCOM at 0.7 significance cut-off are represented in underlined.


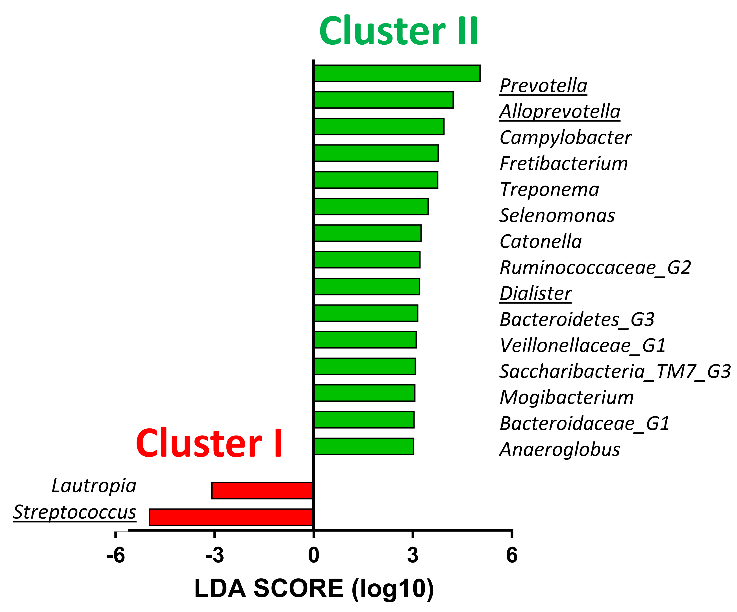


**Supplementary figure 2. Salivary microbiota composition is different between the two clusters.** Bar-plot representing the differentially abundant genera between cluster 1 and cluster 2 inferred by LEfSe at a significance cut-off of 3. The Differential OTUs inferred by ANCOM are represented in underlined at 0.7 significance cut-off.
